# Age-related increases in amyloid beta and membrane attack complex: evidence of inflammasome activation in the rodent eye

**DOI:** 10.1186/s12974-015-0337-1

**Published:** 2015-06-24

**Authors:** Tom Zhao, Jiangyuan Gao, Jenifer Van, Eleanor To, Aikun Wang, Sijia Cao, Jing Z. Cui, Jian-Ping Guo, Moonhee Lee, Patrick L. McGeer, Joanne A. Matsubara

**Affiliations:** Department of Ophthalmology and Visual Sciences, Faculty of Medicine, University of British Columbia, 2550 Willow Street, Vancouver, V5Z 3N9 BC Canada; Kinsmen Lab of Neurological Research, University of British Columbia, Vancouver, BC Canada

**Keywords:** Age-related macular degeneration, Membrane attack complex, Amyloid beta, NLRP3 inflammasome, NF-κB, RPE/choroid

## Abstract

**Background:**

The membrane attack complex (MAC) is a key player in the pathogenesis of age-related macular degeneration (AMD) and is a putative activator of the NLRP3 inflammasome. Amyloid beta (Aβ), a component of drusen deposits, has also been implicated in inflammasome activation by our work and those of others. However, the interactions of MAC and Aβ are still poorly understood, especially their roles in aging and retinal degenerative pathologies. Since inflammasome activation may represent a key cellular pathway underlying age-related chronic inflammation in the eye, the purpose of this study is to identify the effects associated with MAC and inflammasome activation in the retinal pigment epithelium (RPE)/choroid and to evaluate the therapeutic merits of MAC suppression.

**Methods:**

Adult Long-Evans rats were divided into treatment and control groups. Treatment groups received oral aurin tricarboxylic acid complex (ATAC), a MAC inhibitor, in drinking-water, and control groups received drinking-water alone (No ATAC). Groups were sacrificed at 7.5 or 11.5 months, after approximately 40 days of ATAC treatment. To study age-related changes of Aβ and MAC in RPE/choroid, naive animals were sacrificed at 2.5, 7.5, and 11.5 months. Eye tissues underwent immunohistochemistry and western blot analysis for MAC, Aβ, NF-κB activation, as well as cleaved caspase-1 and IL-18. Vitreal samples were collected and assessed by multiplex assays for secreted levels of IL-18 and IL-1β. Statistical analyses were performed, and significance level was set at *p* ≤ 0.05.

**Results:**

In vivo studies demonstrated an age-dependent increase in MAC, Aβ, and NF-κB activation in the RPE/choroid. Systemic ATAC resulted in a prominent reduction in MAC formation and a concomitant reduction in inflammasome activation measured by cleaved caspase-1 and secreted levels of IL-18 and IL-1β, but not in NF-κB activation. In vitro studies demonstrated Aβ-induced MAC formation on RPE cells.

**Conclusions:**

Age-dependent increases in Aβ and MAC are present in the rodent outer retina. Our results suggest that suppressing MAC formation and subsequent inflammasome activation in the RPE/choroid may reduce chronic low-grade inflammation associated with IL-18 and IL-1β in the outer retina.

**Electronic supplementary material:**

The online version of this article (doi:10.1186/s12974-015-0337-1) contains supplementary material, which is available to authorized users.

## Introduction

Age-related macular degeneration (AMD), the leading cause of irreversible blindness in the elderly in developed countries, is a condition of the central retina, characterized by retinal pigment epithelium (RPE) atrophy and photoreceptor loss. Advanced chronological age is an important risk factor for AMD. In North America, the prevalence of AMD increases dramatically with age from 10 % in individuals at 55 to 65 years of age, to 30 % at 75 to 85 years of age [1]. Key to understanding the effects of aging on the pathogenesis of retinal degenerative diseases are the cellular pathways that become dysregulated with age [2–4].

For example, aging is associated with the dysregulation in the complement cascade, part of the innate immune response. The complement cascade causes opsonization and agglutination, as well as cell lysis by the formation of the membrane attack complex (MAC) [5]. Genetic studies showed that certain variants of the complement factor H (CFH) gene, an inhibitor of the alternative pathway, can increase the risk of AMD by up to sixfold in patients with the at-risk variant [6]. Postmortem eyes genotyped for CFH at-risk variants have increased MAC levels in the RPE/choroid [7, 8]. Moreover, in dry AMD patients, those with a CFH Y402H variant have elevated systemic levels of interleukin-6 (IL-6), tumor necrosis factor (TNF-α), and IL-18 [9]. In addition to genetic influences, aging also contributes to the dysregulation of the complement system, as evidenced by the association of activated complement products and increased MAC deposition in the RPE/choroid with advanced age [10–13].

While MAC deposition may cause cell lysis, it may also occur at sublytic levels that promote chronic, low-grade pro-inflammation. This type of chronic, local inflammation in the outer retina has been hypothesized to lead to degenerative changes in RPE function [14]. Recent studies have suggested that MAC may mediate activation of the NLRP3 inflammasome [15, 16]. The inflammasome is a multi-protein complex that activates caspase-1 and produces the pro-inflammatory cytokines IL-1β and IL-18, which in turn have been linked to RPE atrophy [17, 18]. Whether MAC promotes inflammasome activation in the RPE/choroid is not known and is the premise of this study. We postulate that sublytic MAC increases with age, promotes activation of the inflammasome, and thereby, dysregulates RPE function. To ameliorate MAC-induced inflammasome activation on RPE, we also explore the effects of an agent, aurin tricarboxylic acid complex (ATAC), which has been shown to inhibit MAC formation in mice and humans. Specifically, ATAC acts both to inhibit the formation of the C3 convertase and to block the addition of C9 to the C5b-8 complex and thereby inhibits MAC [19, 20]. Here, we examine the age-related increase in MAC, the efficacy of ATAC in lowering levels of sublytic MAC, and a subsequent, corresponding reduction in inflammasome activation in the rat RPE/choroid.

## Methods

### In vivo studies

The animal procedures were approved by the Animal Care Committee of the University of British Columbia, conformed to the guidelines of the Canadian Council on Animal Care and were in accordance with the Resolution on the Use of Animals in Research of the Association of Research in Vision and Ophthalmology. Adult Long-Evans rats (Charles River, Wilmington MA) were divided into four groups. Group 1 (*N* = 6) comprised 6-month-old rats treated with oral administration of ATAC for 40 days and sacrificed at the age of 7.5 months. A dosage of 60 mg/100 mL (in drinking-water) was chosen based on efficacy observed in an earlier study [19]. Group 2 (*N* = 6) comprised untreated 6-month-old rats (controls) sacrificed at the age of 7.5 months. Group 3 (*N* = 6) comprised 10-month-old rats treated with ATAC in drinking-water (60 mg/100 mL) for 40 days and sacrificed at the age of 11.5 months. Group 4 (*N* = 6) comprised untreated 10-month-old rats (controls) sacrificed at 11.5 months. Additional untreated naive animals were sacrificed at 2.5, 6, 7.5, or 11.5 months, and retinal tissues were used to demonstrate age-related changes in MAC, Aβ, and NF-κB activation. At the study endpoints, animals were anesthetized, whole blood drawn for serum analysis, and then euthanized. Eyes were immediately enucleated and frozen (western blot and ELISA) or fixed in 4 % paraformaldehyde in Dulbecco’s phosphate-buffered saline (Invitrogen, Carlsbad CA) for 48–72 h prior to embedding in paraffin.

### Fibrillar Aβ_1–40_ preparation

The lyophilized, synthetic Aβ_1–40_ peptide in its HCl salt form was purchased from American Peptide (Sunnyvale, CA). We chose Aβ_1–40_ peptide over its structurally similar but more toxic, Alzheimer’s disease-specific, form of Aβ_1–42_ peptide based on earlier studies that demonstrated the presence of Aβ_1–40_ in drusen deposits in postmortem human eyes and its effects on upregulation of complement genes in RPE cells in vitro [21, 22]. Fibrillar Aβ_1–40_ was prepared according to published protocols [23, 24]. Briefly, the synthetic Aβ_1–40_ peptide was reconstituted in sterile distilled H_2_O and incubated at room temperature for 30 min, then evaporated by speed vacuum for 1.5 h resulting in thin transparent Aβ_1–40_ peptide film. Aβ_1–40_ peptide film was reconstituted in 100 % dimethyl sulfoxide to a concentration of 1 mM, and further diluted in Tris-buffered saline (TBS; 20 mM Tris-HCl, 100 mM NaCl, pH 7.4, 37 °C) to produce the Aβ_1–40_ stock solution of 100 μM. A longitudinal incubation assay consisting of six sampling time points at 37 °C (24 h to 2 weeks) was set up to optimize the conditions for fibrillar Aβ_1–40_. Based on the protein separation, the sample at 1-week incubation time was selected, due to its higher fibrillar and lower oligomeric contents, which was then further diluted to 0.3 μM (final concentration) for the in vitro stimulation studies (Fig. 2) [22, 25–27].

### In vitro complement activation assay on ARPE-19 cells

An in vitro assay was used to assess whether the fibrillar Aβ_1–40_ induces MAC formation and deposition on ARPE-19 cells. Briefly, cells were seeded and cultured in 8-chambered glass slides. Normal human serum (NHS) (25 %) (CompTech, Tyler, TX) was added to the cell cultures with 0.3 μM fibrillar Aβ_1–40_ for 24 h. Positive controls included zymosan stimulation (10 μg/ml, Sigma Aldrich, St. Louis MO) with 25 % NHS. Negative controls included 25 % heat-inactivated NHS (HI-NHS) with fibrillar Aβ_1–40_ or zymosan, and serum-free medium with 25 % NHS or HI-NHS. MAC formation was detected by immunocytochemistry (Table 1) and quantified as the percentage of RPE cells positive for MAC compared to the total number of cells per × 40 field. The experiments were done in hexaplicates for each stimulation condition (*N* = 6).

**Table 1:** List of primary antibodies

Antigen	Antibody	Dilution	Source	Applications
Membrane attack complex (MAC)	Mouse monoclonal (clone aE11)	1:50 (IHC) 1:1000 (WB)	Dako, Burlington, ON, Canada	Immunocytochemistry Western blot
Membrane attack complex (MAC)	Rabbit polyclonal	1:500	BIoss, Woburn, MA	Immunohistochemistry
Interleukin-18 (IL-18)	Rabbit polyclonal	1:100	Santa Cruz Biotechnology, Dallas, TX	Immunohistochemistry
Amyloid-beta amino acid 17–24 (Aβ_17–24_)	Mouse monoclonal anti-Aβ_17–24_ (clone 4G8)	1:400 (IHC) 1:1000 (WB)	BioLegend, Dedham, MA	Immunohistochemistry Western blot
Phosphorylated NF-κB p65 (Ser 276)	Rabbit polyclonal	1:75 (IHC) 1:500 (WB)	Santa Cruz Biotechnology, Dallas, TX	Immunohistochemistry Western blot
Phosphorylated NF-κB p50 (Ser 337)	Rabbit polyclonal	1:500 (WB)	Santa Cruz Biotechnology, Dallas, TX	Western blot
Caspase-1	Mouse monoclonal	1:1000	R&D Systems, Minneapolis, MN	Western blot
Amyloid-beta amino acid 1–16 (Aβ_1–16_)	Mouse monoclonal anti-Aβ_1–16_ (clone 6E10)	1:2000	BioLegend, Dedham, MA	Western blot
GAPDH	Mouse monoclonal	1:10,000	EMD Millipore, Billerica, MA	Western blot

### Quantification of ATAC and CH50 assay

ATAC synthesis and its serum concentration analysis followed published methods [19, 20]. The final ATAC concentration was expressed in μg per 500 μL of blood (*N* = 6). A CH50 hemolysis assay was used to assess complement activation in rat serum before and after ATAC administration, following published procedures [19].

### Immunohistochemistry (MAC, IL-18, Aβ, and NF-κB)

Paraffin-embedded eye tissues were prepared and sectioned following established protocols [28]. Sections from the paired groups (7.5 m ATAC and No ATAC; 11.5 m ATAC and No ATAC) and the three age groups (2.5, 7.5, and 11.5 m) were processed simultaneously in order to make intensity comparisons. Primary antibodies targeting MAC, IL-18, and Aβ are described in Table 1. For the negative control sections, the primary antibody was replaced with a matched non-specific isotype IgG (Sigma Aldrich). For visualization, the slides were developed using either the Vector® VIP substrate kit or Vector® AEC substrate kit.

**Fig. 1:**
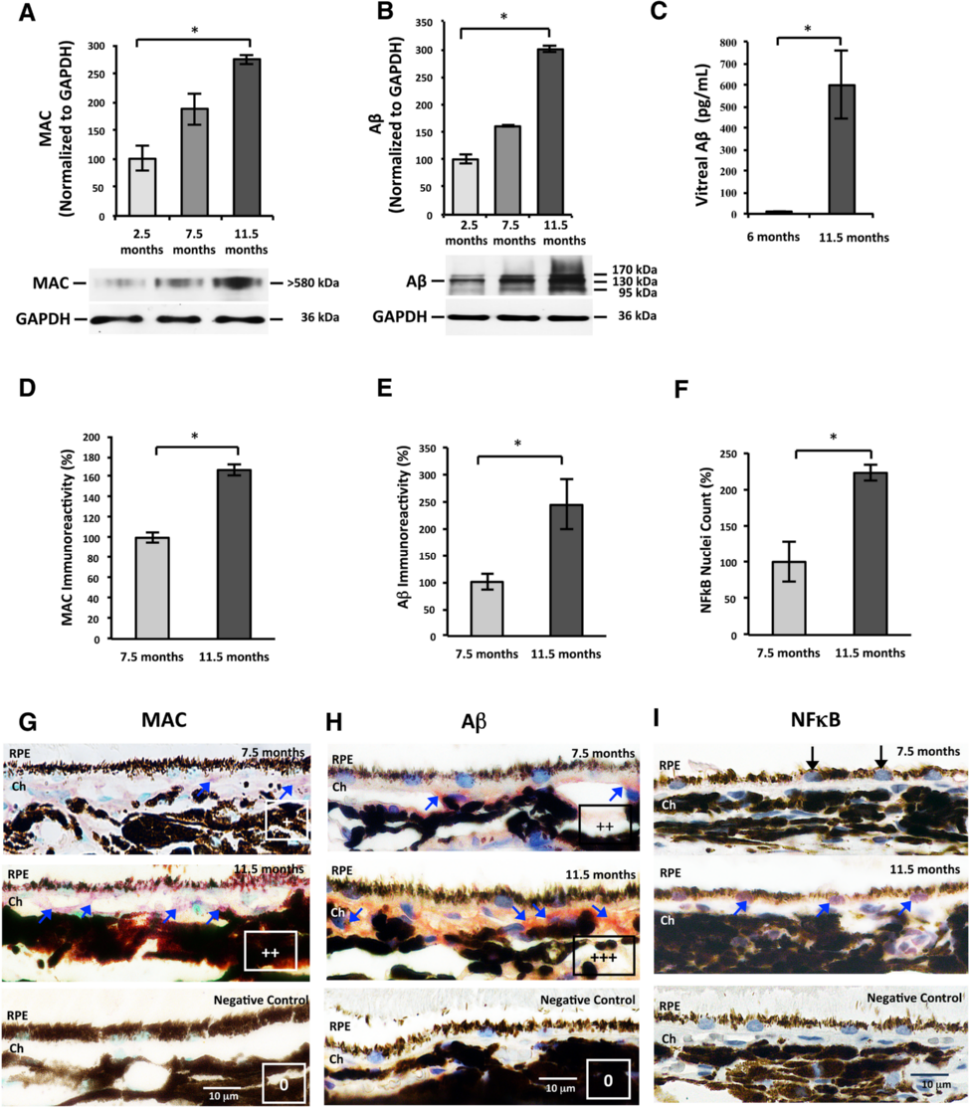
Age-dependent increases of ocular MAC, Aβ and NF-κB. **a** Western blot analysis revealed increasing MAC levels in the rat RPE/choroid with age from 2.5 to 11.5 months old (Kruskal-Wallis, *p* ≤ 0.05). **b** Western blot analysis showed enhanced accumulation of high-molecular weight Aβ (MW > 95 kDa) in the rat RPE/choroid with age from 2.5 to 11.5 months old (Kruskal-Wallis, *p* ≤ 0.05). **c** ELISA measurements showed a dramatic increase in the soluble Aβ levels in the vitreous fluids of the 11.5 months old (599.1 pg/mL) compared to the 6 months old group (7.5 pg/mL) (Mann-Whitney, *p* ≤ 0.05). **d**, **e** Analysis of MAC deposition (**d**) or Aβ (**e**) in rat RPE/choroid demonstrated a significant increase in immunoreactivity with increasing age, with data normalized to the younger age group of 7.5 months (Mann-Whitney, *p* ≤ 0.05). **f** NF-κB activation in RPE increased with age. The percentage of RPE cells with nuclear labeling of translocated NF-κB p65 subunit was higher in the retina of the 11.5 months old group compared to the 7.5 months old group (Mann-Whitney, *p* ≤ 0.05). **g** Representative micrographs of MAC immunoreactivity at both ages (7.5 and 11.5 months) showed MAC deposition on the basal side of RPE and in choroid. Immunoreactivity was processed with VIP, resulting in a purple color (*blue arrows*) and nuclei counterstained with Methyl Green. Background immunoreactivity (0) and semi-quantitative scoring of + and ++ are given for the following examples: 7.5 months old (+), 11.5 months old (++), and negative control (0). Note the dark brown choroidal melanocytes in the 11.5 months picture appeared surrounded by red haze due to bright field illumination. **h** Representative micrographs of Aβ immunoreactivity at both ages (7.5 and 11.5 months) showed positive immunolabeling on the basal side of RPE and in choroid. Immunoreactivity was processed with AEC, resulting in a red color (*blue arrows*) and nuclei counterstained as blue. Examples of semi-quantitative scores are given as follows: 7.5 months old (++), 11.5 months old (+++), and negative control (0). **i** Representative micrographs of the RPE nuclei from the 11.5 months old group showed more robust NF-κB p65 immunoreactivity than the 7.5 months old group. Positive NF-κB p65 immunolabeling is purple in RPE nuclei and indicated by *blue arrows*. RPE nuclei devoid of NF-κB p65 are blue and indicated by *black arrows*. Scale bars; 10 μm. *RPE* retinal pigment epithelium, *Ch* choroid

MAC, Aβ, and IL-18 immunoreactivity was scored in a masked fashion and semi-quantitatively based on a 0–3 point scale (see Fig. 1 legend). Analysis and micrographs were taken using a × 60 objective lens and × 10 eyepieces (*N* = 3). The immunoreactivity scores of MAC, Aβ, and IL-18 were averaged and normalized to the 7.5-month-old group or the untreated control group (No ATAC).

To detect the active NF-κB, an antibody against the NF-κB p65 subunit was used (Table 1). Immunoreactivity was scored quantitatively, in a masked fashion, using a × 60 objective lens and × 10 eyepieces (*N* = 3). Positive RPE nuclei were identified as containing both the red (AEC) chromogen and blue hematoxylin counterstain, thus resulting in a purple appearance distinct from the unlabeled RPE nuclei that were blue in color from only the hematoxylin counterstain. The number of NF-κB positive nuclei was converted to percentage of all RPE nuclei in the sample area, and normalized to the untreated control group (No ATAC) or the 7.5-month-old group.

### Western blot

RPE/Bruch’s membrane (BM)/choroid tissues were isolated and pooled for animals in each of the four treatment groups (groups 1–4, *N* = 6) and in each age group of the naive animals (2.5, 7.5, and 11.5 months; *N* ≥ 3). To detect the MAC deposits, the tissue samples were homogenized in 200 μL of ice-cold MAC extraction buffer (50 mM Tris-HCl (pH 6.8), 150 mM NaCl, 0.1 % SDS) containing protease inhibitor cocktail (Roche Diagnostics, Indianapolis IN) [20]. To preserve the MAC protein complex, 40 μg of total protein was mixed with equal volume of 2× non-reducing loading buffer, devoid of boiling, and directly subjected to 5–10 % SDS-PAGE. Proteins were transferred to a PVDF membrane and incubated with a series of blocking buffers, primary antibody against MAC (Table 1), and HRP conjugated secondary antibody (R&D Systems, Minneapolis, MN). The enhanced chemiluminescence (ECL) method was used to detect the MAC protein bands from animal groups 1–4 and the naive animals at three different ages. The glyceraldehyde 3-phosphate dehydrogenase (GAPDH)-loading control blot was done similarly using a mouse GAPDH antibody (Table 1). All protein bands were subsequently quantified using Image J (NIH, Bethesda MD), and the ratio of MAC-to-GAPDH was calculated. The final relative intensity of MAC was normalized either to the youngest age group of 2.5 months or to the No ATAC control group.

To detect caspase-1 cleavage and NF-κB activation, the RPE/BM/choroid tissues were homogenized in 200 μL of ice-cold RIPA buffer (Thermo Scientific, Waltham, MA) containing protease inhibitor cocktail (Roche Diagnostics). Blotting procedures followed our established protocol [28]. For GAPDH, the same membrane was incubated in stripping buffer and then re-probed with the GAPDH antibody (Table 1). The protein band intensity of cleaved caspase-1 (20kD), phosphorylated NF-κB p65 subunit (65 KDa), phosphorylated NF-κB p50 subunit (50 KDa), and GAPDH (36kD) was individually measured using Image J and converted into ratios relative to GAPDH. The final relative intensity of cleaved caspase-1 p20 and phosphorylated NF-κB p65 was normalized to the No ATAC control group.

For western blot detection of Aβ, both fibrillar Aβ_1–40_ preparation and the RIPA buffer-extracted RPE/BM/choroid tissue lysates were mixed with 2× non-reducing loading buffer, devoid of boiling, and directly subjected to 5–12 % SDS-free PAGE separation. Electrophoresis was run using MES buffer (Invitrogen, pH 7.3 ~ 7.7), and proteins were transferred onto a 0.2-μm PVDF membrane. The anti-Aβ 6E10 antibody was used to detect the fibrillar Aβ_1–40_ preparation, whereas the anti-Aβ 4G8 antibody was used for tissue lysates (Table 1). Aβ bands were developed by the ECL method. For the membrane containing tissue lysate proteins, it was stripped and re-processed for GAPDH detection. The intensity of high-molecular species Aβ (MW > 95 kDa) and GAPDH (36kD) was independently measured using Image J and converted into ratios of Aβ-to-GAPDH. The final relative intensity ratio of Aβ-to-GAPDH was normalized to the youngest age group of 2.5 months.

### ELISA for Aβ

A chemiluminescent ELISA assay specific for the detection of Aβ x-40 isoform was used to quantify Aβ in the vitreous of rat eyes (BioLegend, Dedham, MA). 6-month-old (*N* = 4) and 11.5-month-old (*N* = 6) rats were sacrificed for vitreous collection. Vitreous samples were diluted with the HRP detection antibody at a ratio of 1:1. After 18 h of detection antibody incubation at 4 °C, the ELISA plate was then incubated with chemiluminescent substrates for 15 s and imaged with a microplate reader (Synergy H1, BioTek, Winooski, VT). A non-linear 4-parameter regression model was used to generate the standard curve to calculate Aβ concentrations of all vitreous samples (Gen5 version 2.04.11, BioTek).

### Suspension array for IL-1β and IL-18

An ELISA-based cytokine assay for the mature, secreted products of the inflammasome, IL-1β and IL-18, was carried out (Bio-Plex 200 System, Bio-Rad Laboratories, Hercules CA). Vitreous from rat eyes in groups 3–4 were pooled. Experiments were carried out following methods in our earlier publication [28].

### Statistical analyses

**Fig. 2:**
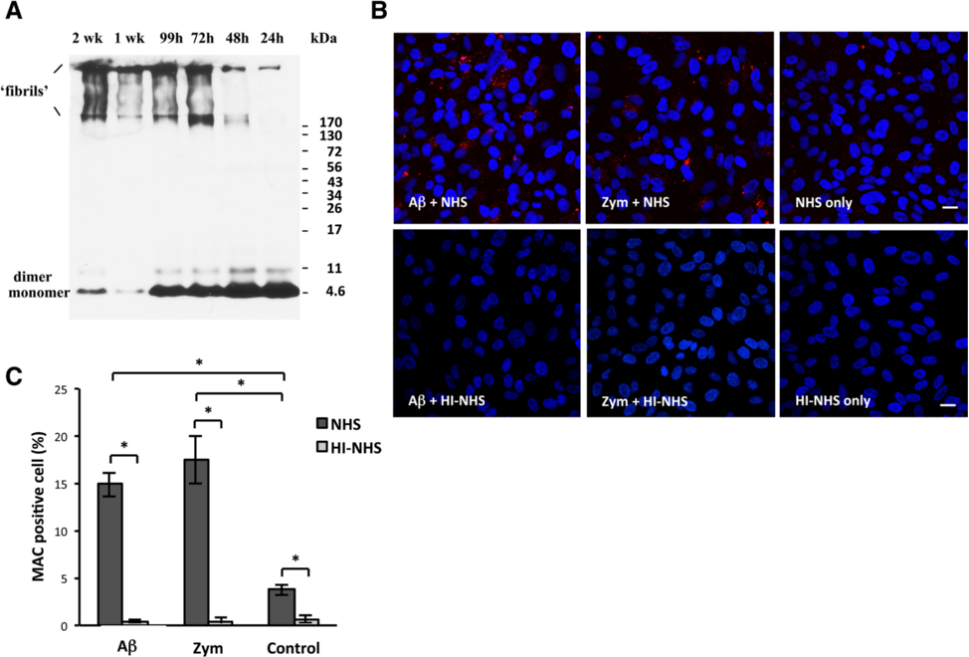
Aβ induces MAC deposition on RPE in vitro. **a** Western blot of fibrillar Aβ_1–40_ preparation from six sampling time points. A general increase of high-molecular weight, fibrillar Aβ_1–40_ (MW > 170 kDa), and a concomitant decrease of low-molecular weight Aβ_1–40_ (MW < 17 kDa) are seen with increasing incubation time. Among all the time points, the 1-week incubation yielded a relatively higher percentage of fibrillar Aβ_1–40_ compared to monomeric and dimeric species than the other time points. **b** Representative confocal microscopic images showed significantly more MAC deposits (Cy3, *red*) on ARPE-19 cells by combinatorial stimulation of fibrillar Aβ_1–40_/NHS or zymosan (Zym)/NHS than by NHS alone. HI-NHS replacement abolished MAC deposition on RPE cells regardless the presence of fibrillar Aβ_1–40_ or Zym. Scale bars; 20 μm. **c** Stimulation with fibrillar Aβ_1–40_ or Zym (positive control) in the presence of NHS resulted in a greater percentage of MAC-labeled RPE cells than in the presence of HI-NHS (Mann-Whitney, *p* ≤ 0.05). The *control bars* indicate background level of MAC labeling as demonstrated by incubating ARPE19 cells with NHS, and this resulted in higher labeling than in HI-NHS (Mann-Whitney, *p* ≤ 0.05). Comparisons between the three groups, fibrillar Aβ_1–40_, zymosan, and control, in the presence of NHS, demonstrated significance between fibrillar Aβ_1–40_ and control, as well as between zymosan and control but not between fibrillar Aβ_1–40_ and zymosan (Kruskal-Wallis, *p* ≤ 0.05)

**Fig. 3:**
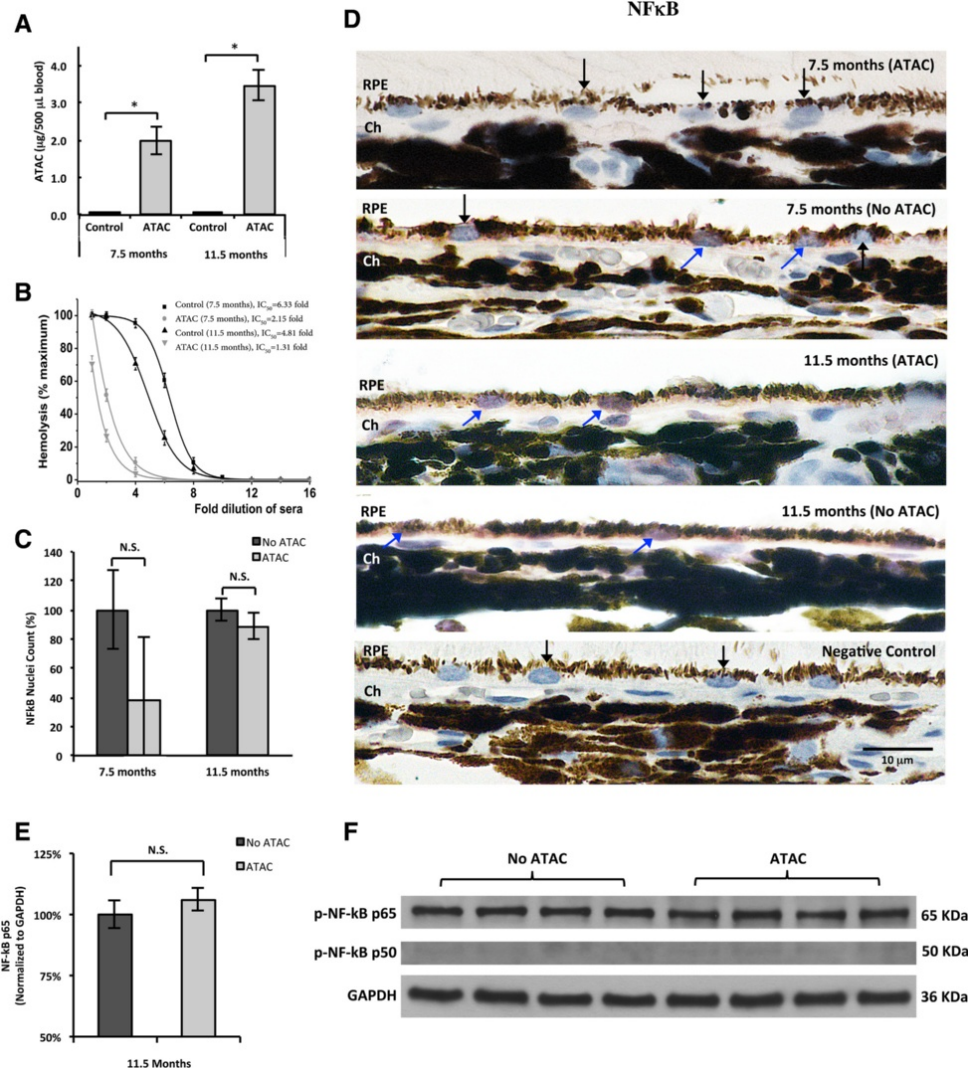
Systemic ATAC administration did not alter NF-κB activation in RPE. **a** The amount of ATAC in blood was measured after 40 days of drug administration in 7.5-month-old (1.98 μg/500 μL) and in 11.5-month-old (3.5 μg/500 μL) animals. Animals treated with ATAC showed significantly higher ATAC blood concentrations than age-matched controls without ATAC treatment at both ages (Mann-Whitney, *p* ≤ 0.05). **b** The degree of inhibition on total complement activation by ATAC was measured by a CH50 hemolysis assay. Dilutions of the sera containing different levels of ATAC were performed to calculate the half maximal inhibitory concentration (IC_50_) for total complement activity. Higher IC_50_ values proportionally reflect higher levels of complement activity. Note that the IC_50_ levels are lower (i.e., *curves shifted to the left*) for ATAC-treated animals compared to untreated controls at both ages. **c** ATAC administration did not affect NF-κB activation in RPE at both ages of 7.5 and 11.5 months (Mann-Whitney, *p* > 0.05). **d** Representative micrographs of NF-κB p65 immunoreactivity in RPE/choroid from each group in **c**. RPE cells that demonstrate NF-κB p65 nuclear translocalization have purple nuclei and are marked by *blue arrows*. Unlabeled RPE nuclei are counterstained with hematoxylin (*blue only*) and are marked by *black arrows*. Scale bar; 10 μm. *RPE* retinal pigment epithelium, *Ch* choroid. **e**, **f** RPE/choroid tissue lysates from 11.5-month-old rats with ATAC administration contained the same amount of phosphorylated p65 subunit as in rats without ATAC in drinking-water (Mann-Whitney, *p* > 0.05). The level of phosphorylated p50, however, was extremely low in both groups

**Fig. 4:**
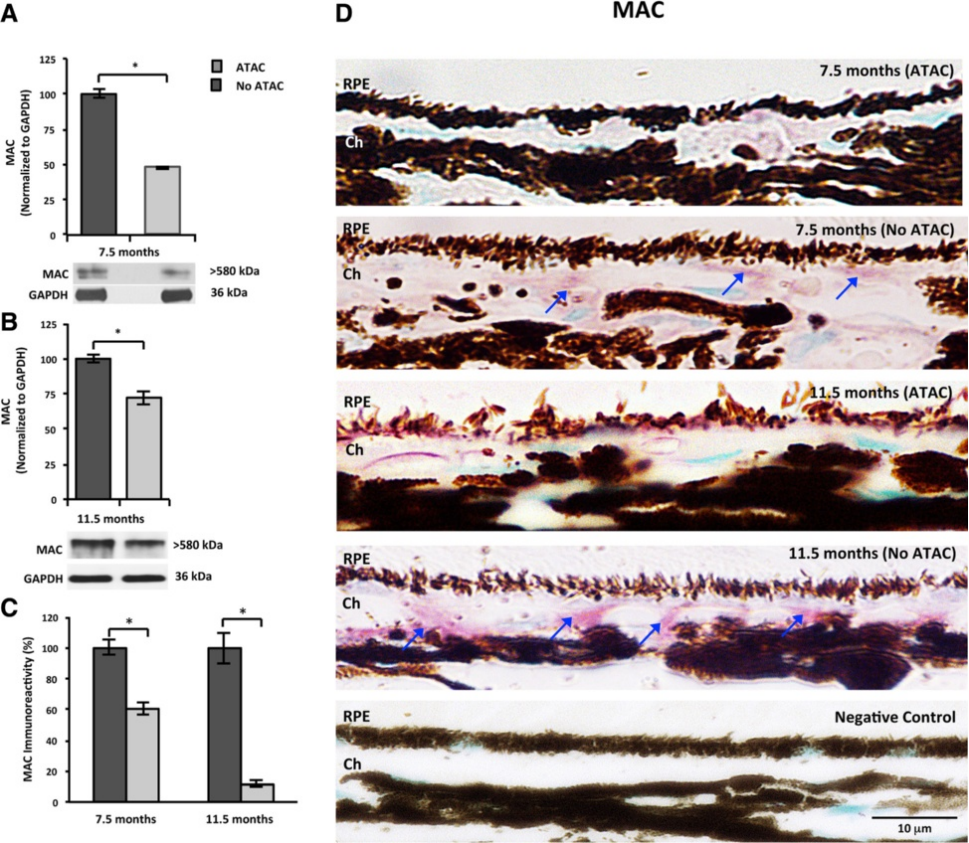
ATAC treatment suppresses MAC deposition in the RPE/choroid. **a**, **b** At both ages of 7.5 months (**a**) and 11.5 months (**b**), western blot analysis showed that ATAC significantly reduced MAC deposits in rat RPE/choroid (Mann-Whitney, *p* ≤ 0.05). **c** At both ages, MAC immunoreactivity in the rat RPE/choroid was significantly lower in the ATAC-treated group compared to the age-matched, No ATAC control group (Mann-Whitney, *p* ≤ 0.05). **d** Representative micrographs from each group in **c** showed MAC immunoreactivity on the basal side of RPE cells and in choroid. *Blue arrows* identify positive MAC deposits labeled with VIP chromogen (*purple*). Nuclei were counterstained as *green*. Scale bar; 10 μm. *RPE* retinal pigment epithelium, *Ch* choroid

**Fig. 5:**
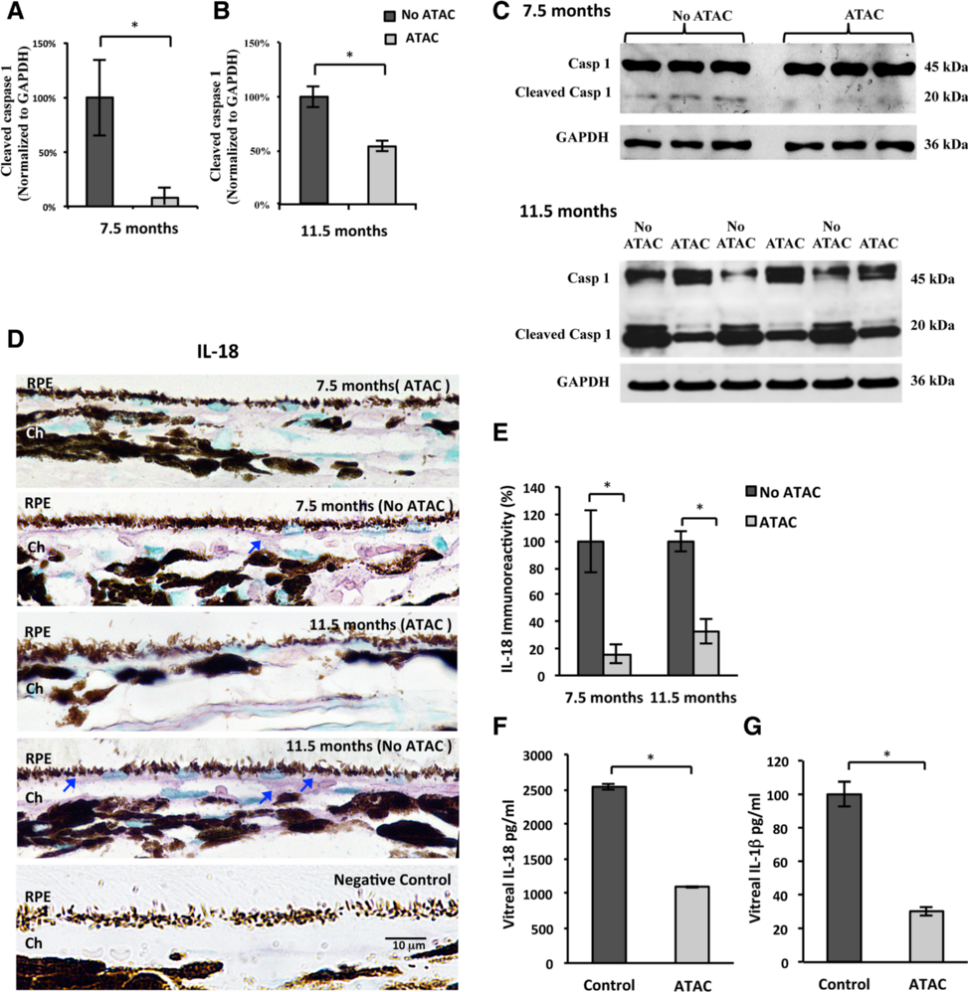
ATAC treatment inhibited inflammasome activation in the RPE/choroid. **a**–**c** At both ages of 7.5 months (**a**) and 11.5 months (**b**), western blot analysis showed that ATAC significantly inhibited pro-caspase-1 (MW 45 kDa) cleavage into active caspase-1 (MW 20 kDa) in rat RPE/choroid (Mann-Whitney, *p* ≤ 0.05). **c** Images of western blot demonstrates a concomitant increase in the pro-caspase-1 band and decrease in the active caspase-1 band after ATAC treatment in both age groups. **d** Representative micrographs illustrate immunoreactivity for IL-18, a product of inflammasome activation, on the basal side of RPE cells and in choroid from treated and untreated animals at 7.5 and 11.5 months of age. *Blue arrows* indicate positive IL-18 labeling (VIP, *purple*). Scale bar; 10 μm. *RPE* retinal pigment epithelium, *Ch* choroid. **e** IL-18 immunoreactivity in the rat RPE/choroid was significantly downregulated by ATAC treatment. Labeling was normalized to 100 % for the untreated animals in each age group (Mann-Whitney, *p* ≤ 0.05). **f**, **g** ELISA measurements of vitreous samples taken from treated and untreated control animals at 11.5 months of age. Note the dramatic reduction in secreted IL-18 (**f**) and IL-1β (**g**) concentrations after ATAC treatment (Mann-Whitney, *p* ≤ 0.05)

Non-parametric tests were used throughout the study. For the two group comparisons (Figs. 1c–f, 2c, 3a, c, e, 4a–c, and 5a, b, e–g), a Mann-Whitney *U* test (one-tailed) was used. For the three group comparisons, a Kruskal-Wallis and post hoc Dunn’s multiple comparisons test was used to determine differences among age groups (Fig. 1a, b) or among stimulation regimens (Fig. 2c). All analyses were conducted with GraphPad Prism version 6.00 for Windows (GraphPad Software, La Jolla, CA). Statistical significance was set at *p* ≤ 0.05.

## Results

### Age-dependent increases of MAC, Aβ, and NF-κB in the RPE/choroid

In this study, we first asked whether MAC deposits increase in normal aging and, if so, is it related to inflammasome activation in the RPE/choroid. A significant increase in MAC (MW > 580 kDa) was evident in the RPE/choroid homogenates of rats ranging in age from 2.5 to 11.5 months using western blot. The normalized MAC levels were 1.88-fold higher at 7.5 months and 2.75-fold higher at 11.5 months when compared to the samples obtained from 2.5-month-old rats (Fig. 1a).

Aβ is a known pathological activator of complement cascade in Alzheimer’s disease (AD) [29]. Its ocular presence has been reported in drusen of postmortem eyes [30] and in rodent eyes [31]. To correlate Aβ accumulation with MAC formation, we semi-quantitatively compared the levels of high-molecular weight Aβ species (MW > 95 kDa) among the RPE/choroid homogenates from different ages. We found an age-dependent increase of high-molecular weight Aβ from 2.5 to 11.5 months (Fig. 1b). Based on the knowledge that biosynthesized Aβ is present in both retina and the vitreous compartment of the eye [32], we quantified the Aβ in rat vitreous samples at two ages. With increasing age, the vitreal Aβ concentration increased, by almost 80-fold, from 7.49 ± 5.16 pg/mL at 6 months to 599.10 ± 159.25 pg/mL at 11.5 months of age (Fig. 1c).

To support these results, we also assessed MAC formation and Aβ accumulation in retinal cross sections by immunohistochemistry. We demonstrated that there are increasingly higher levels of immunoreactivity of both MAC and Aβ in the 11.5 month animals compared to the 7.5-month-old animals, particularly in the choroid and the basal side of RPE (Fig. 1d, e, g, h).

NF-κB is a major transcription factor that responds to a variety of pro-inflammatory signals by nuclear translocalization to upregulate the expression of target genes. Previously, we demonstrated that Aβ activates NF-κB, which can be specifically inhibited by NF-κB antagonists (e.g., vinpocetine or BAY 11–7082) [28]. In the present study, by using an antibody targeting the phosphorylated p65 subunit of the translocated NF-κB, we observed an increase in the percentage of RPE nuclei harboring the phosphorylated p65 subunit, mirroring the age-related increase we observed with both MAC and Aβ (Fig. 1f, i).

### Aβ promotes MAC formation in cultured ARPE-19 cells

The AD literature suggests that Aβ binds to C1q and thereby promotes the classic complement cascade [29]. To further understand the relationship between MAC and Aβ in the RPE/choroid, we undertook stimulation studies to assess MAC formation on RPE in vitro. Cells were stimulated with 0.3-μM fibrillar Aβ_1–40_ (MW > 170 kDa), which was prepared from synthetic Aβ_1–40_ peptide and tested by western blot. In the presence of 25 % NHS, we observed MAC deposition on cells as ascertained by immunocytochemistry with an anti-MAC antibody and imaged by confocal microscopy. MAC formation was prevented in control experiments in which cells were incubated with 25 % HI-NHS. Levels of MAC resulting from fibrillar Aβ_1–40_-stimulation were compared to MAC deposition on cells treated with zymosan (positive control) or serum-free medium (negative control) in the presence of NHS or HI-NHS. NHS alone resulted in 3.73 % of RPE cells positive for MAC. However, when combined with fibrillar Aβ_1–40_ or zymosan, MAC deposition increased to 14.91 % and 17.52 % of total RPE cells counted, respectively (Fig. 2).

### ATAC did not affect local NF-κB activation in RPE

While ATAC inhibits complement cascade, it is unclear whether systemic administration of ATAC would reduce local inflammation in the eye. To test this, animals were treated with oral administration of ATAC in drinking-water (60 mg/100 mL) for 40 days. Animals readily accepted drinking-water laced with ATAC ad libitum, and no overt signs of side effects or toxicity were noted, consistent with our earlier studies in which ATAC was given in food [19].

After 40 days, the average ATAC level, as measured by fluorescence spectroscopy, was 1.98 and 3.5 μg/500 μL in the 7.5 and 11.5-month-old ATAC-treated rats, respectively. As expected, no appreciable amount of ATAC was evident in control rats at either age group (Fig. 3a). A CH50 assay, which measures the amount of hemolysis in blood due to complement activation, has been used before as a surrogate marker for complement activity both clinically and in experimental studies [19, 33]. Our results showed that sera from the ATAC-treated animals displayed a significant three- to fourfold decrease in hemolysis relative to the sera of the control animals in both age groups studied, thus confirming that orally administered ATAC was present and effective at inhibiting complement activation in the sera of treated rats (Fig. 3b). Next, we assessed the nuclear translocation of the p65 subunit in RPE after ATAC treatment. Intriguingly, systemic administration of ATAC did not change NF-κB p65 nuclear translocation when compared to control animals at 7.5 or 11.5 months old (Fig. 3c, d). Further western blot analyses of phosphorylated NF-κB p65 and p50 subunits in 11.5-month-old rats supported this, and no significant difference in normalized band intensity was found between animals receiving ATAC or drinking-water (Fig. 3e, f).

### ATAC reduced MAC deposition in the RPE/choroid

Our earlier studies showed that ATAC was effective at decreasing MAC in the central nervous system [19, 20], and our next question was whether the observed systemic level of ATAC was sufficient to inhibit MAC formation locally in the eye. To answer this, ATAC-treated animals (7.5 and 11.5 months old) were sacrificed, and tissue lysate of RPE, choroid, and BM underwent western blot analysis. At both ages, animals treated with ATAC demonstrated less MAC deposits (MW > 580 kDa) in the RPE/choroid compared to non-ATAC-treated animals. Furthermore, ATAC treatment was more effective at suppressing MAC in the younger group (7.5 months old). This is intriguing, as the younger group demonstrated a lower ATAC concentration in sera (1.98 μg/500 μL), but with a proportionally greater inhibition of MAC (50 % inhibition) compared to the older group with 3.5 μg/500 μL ATAC in sera, yet only 25 % inhibition of MAC (Figs. 3a and 4a, b). To better understand the distribution of MAC in the ocular compartments after systemic ATAC, we assessed MAC in retinal cross sections by immunohistochemistry. MAC immunoreactivity was robust in the choroid and BM of non-ATAC-treated rats at both ages, and it was reduced by ATAC treatment at both ages (Fig. 4c, d).

### ATAC suppressed inflammasome activation in the RPE/choroid

Earlier studies in non-ocular systems suggest that MAC formation is a potential trigger for inflammasome activation [15, 16]. Here we used an inhibitor of MAC, ATAC, to suppress inflammasome activation in the RPE/choroid. To test whether MAC promotes inflammasome activation, we first examined the level of pro-caspase-1 (MW 45 kDa) cleavage in RPE/choroid homogenates using western blot. At both ages tested (7.5 and 11.5 months), ATAC successfully lowered cleaved caspase-1 (MW 20 kDa) by ~90 % (7.5 months old) and by ~50 % (11.5 months old), when compared to untreated controls (Fig. 5a–c). Next, we assessed the levels of two mature products of inflammasome activation, IL-1β and IL-18. Compared to the ATAC group, we found that IL-18 immunoreactivity was higher in the untreated rat RPE/choroid. On closer visual examination, it was evident that the majority of the IL-18 immunolabeling was located to the basal side of RPE and at the RPE/choroid interface (Fig. 5d, e). We next tested secreted levels of IL-1β and IL-18 in the vitreous by custom-made ELISA assays. Secreted levels of IL-1β and IL-18 were 3- and 2.5-fold lower in the vitreous of animals treated with ATAC, respectively, compared to the untreated age-matched controls (Fig. 5f, g).

## Discussion

With normal aging, the RPE/choroid complex undergoes many changes including drusen deposition, thickening of BM, and thinning of the choroid. The RPE also undergoes a number of age-associated changes, including loss of melanin granules, accumulation of lipofuscin, changes in pro-inflammatory cytokine secretion, and even cell death [34, 35]. However, the cellular mechanisms underlying these changes in the RPE/choroid remain largely unknown. Here, we provide a new perspective by investigating the relationship among aging, MAC formation, and inflammasome activation.

### Aβ facilitates MAC formation and MAC-induced inflammasome activation in the RPE/choroid

In AD, Aβ is a known activator of the classic complement pathway [36, 37]. In the eye, it is also abundant, co-localizes with complement factors in drusen, and demonstrates an age-associated increase [38]. We previously showed by pathway analysis (Ingenuity, GSEA) that the complement system is triggered by Aβ stimulation of RPE in vitro [22]. However, little has been done to assess the potential interaction between Aβ and the complement terminal product, MAC. Here we report age-associated increases in MAC and Aβ in the RPE/choroid complex and soluble Aβ in the vitreous fluids (Fig. 1). The observed age-associated MAC formation in rat RPE/choroid is consistent with our earlier findings of MAC deposition in BM and choroid of older postmortem human eyes [10]. Since MAC and Aβ are located at the interface of RPE and choroid, we assessed MAC formation on RPE in the presence of Aβ and NHS and showed for the first time that fibrillar Aβ is an effective inducer of the complement cascade resulting in MAC deposition (Fig. 2). MAC mediated inflammasome activation was shown previously in cells derived from bone marrow and lung epithelial cells in vitro [15, 16]. Our data support their findings and extend it to an ocular cell type, the RPE.

From our work, and those of others, it is plausible that the inhibition of MAC in the RPE/choroid will dampen inflammasome activation in RPE. We found that ATAC administration concomitantly prevented the full-length caspase-1 from being cleaved into an enzymatically mature caspase-1 p20 subunit, the signature event of inflammasome activation in the same animals in which we observed a reduction in MAC levels (Figs. 4 and 5). However, NF-κB activation, a primer for inflammasome activation, was not affected by ATAC treatment, demonstrated by statistically equivalent amounts of the phosphorylated p65 subunit in both retinal sections and RPE/choroid lysates (Fig. 3). Intriguingly, the phosphorylated p50 subunit’s level was extremely low in both ATAC and drinking-water-treated rats. Although the p50/p65 heterodimer is considered the primary form of NF-κB complex in a wide variety of cell types, there are other active NF-κB dimeric combinations that do not require either one or both of them [39]. All of these suggest that ATAC’s inhibitory effects spare NF-κB activation that involves p65 or p50 and are specific for MAC. Hence, the significant reduction in inflammasome activation products, IL-18 and IL-1β, which we observed after ATAC treatment, is likely due to inefficient post-translational processing by mature caspase-1, rather than due to altered pro-IL-18 and pro-IL-1β production by NF-κB pathway (Fig. 5). Whether and how the specific inhibition of NF-κB pathway can in turn affect MAC formation remains elusive and is beyond the scope of the current study. Although the literature suggests NF-κB signaling regulates multiple genes in the complement cascades, such as CFB, C3, and C4, our data indicates no effects on C5a production when NF-κB activation is blocked by vinpocetine in vivo (see Additional file 1), and thus, likely not affecting MAC formation as well [28, 40–42].

### Age-associated differences in MAC deposition and MAC inhibition by ATAC

Another interesting outcome of our study is the difference in ATAC efficacy in the two age groups tested. The younger rats had lower ATAC levels in blood (~2 μg/500 μL) than the older rats (~3.5 μg/500 μL) (Fig. 3), yet there was a proportionally greater decrease in MAC, caspase-1 cleavage, and secreted IL-18 in the younger rats compared to the older group, suggesting that ATAC treatment was more efficacious in the younger group (Figs. 4 and 5). The exact mechanism behind this finding is not clear. One possible explanation is that, with age, there is more Aβ accumulation in the rat eye, leading to more robust NF-κB activation (p65 nuclear translocalization) and more MAC deposition, which is not proportional to the increase in ATAC concentration, or its activity, and thus overwhelmed ATAC’s suppressive effects leading to reduce efficacy in the older rats compared to that observed in younger rats (Figs. 1 and 2).

## Conclusions

**Fig. 6:**
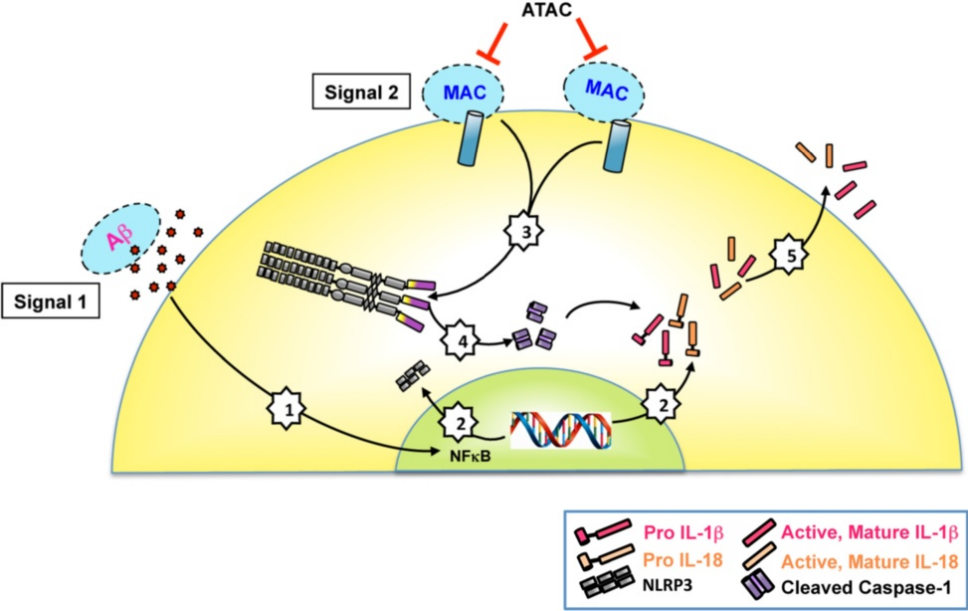
Proposed inflammasome activation mechanisms with Aβ and MAC in RPE/choroid. Age-associated changes in RPE/choroid include an increased accumulation of Aβ that acts as a “Signal 1” to activate the NF-κB pathway (*1*). This upregulates the transcription of NLRP3, pro-IL-18 and pro-IL-1β (*2*). Next, assembly and activation of the NLRP3 inflammasome is triggered by MAC deposition (“Signal 2”) on the RPE cell membrane (*3*). NLRP3 activation results in pro-caspase-1 auto-cleavage (*4*). The cleaved caspase-1 then functions as a cytokine-processing enzyme to facilitate the production and secretion of active, mature IL-18 and IL-1β (*5*). ATAC compound works as a MAC inhibitor, suppressing MAC-induced inflammasome activation on RPE

In summary, we have shown an age-dependent increase in Aβ, MAC, and NF-κB in rat RPE/choroid. We have also demonstrated that Aβ, a drusen component, is an effective priming signal for NF-κB activation in vitro and in vivo [28] and promotes the NLRP3 inflammasome activation in the rat eye [21]. Suppression of MAC leads to a concomitant suppression of NLRP3 inflammasome activation measured by caspase-1 cleavage and secretion of mature IL-18 and IL-1β as depicted in the schematic summary (Fig. 6). An inherent limitation of this study is that rodents do not have a macula/fovea, and thus renders this animal model less useful as it does not reproduce “macular” disease. However, this model is useful to understand the basic, cellular changes in the retina that are associated with chronic inflammation, aging, and age-related retinal diseases. Our work suggests that MAC-induced NLRP3 inflammasome activation may be an important cause of the chronic pro-inflammatory environment in the outer retina of a normal aging eye.

## Additional file

Additional file 1:
**C5a is not affected by Vinpocetine-mediated NF-κB inhibition.** Description of data: Since the intraocular Aβ_1–40_ injection has been previously implicated in promoting NF-κB activation and NLRP3 inflammasome activation [21], we assessed the level of activated C5 (C5a) as a surrogate marker for complement activation and MAC formation when NF-κB activity was specifically inhibited by vinpocetine. Western blot of retina protein lysates shows equal amounts of C5a (MW 41 KDa; rabbit polyclonal C5a complement antibody, cat# 250565) in the vinpocetine-treated group, when compared to vehicle controls (Mann-Whitney, *p* > 0.05; *N* = 5). For a full description of the experimental procedures, readers are referred to our previous publication [28].
