# Supplementary figures and images for: Age-related increases in amyloid beta and membrane attack complex: evidence of inflammasome activation in the rodent eye
Source: J Neuroinflammation. 2015 Jun 24;12:121. doi: 10.1186/s12974-015-0337-1 (PMC4486438; doi:10.1186/s12974-015-0337-1)

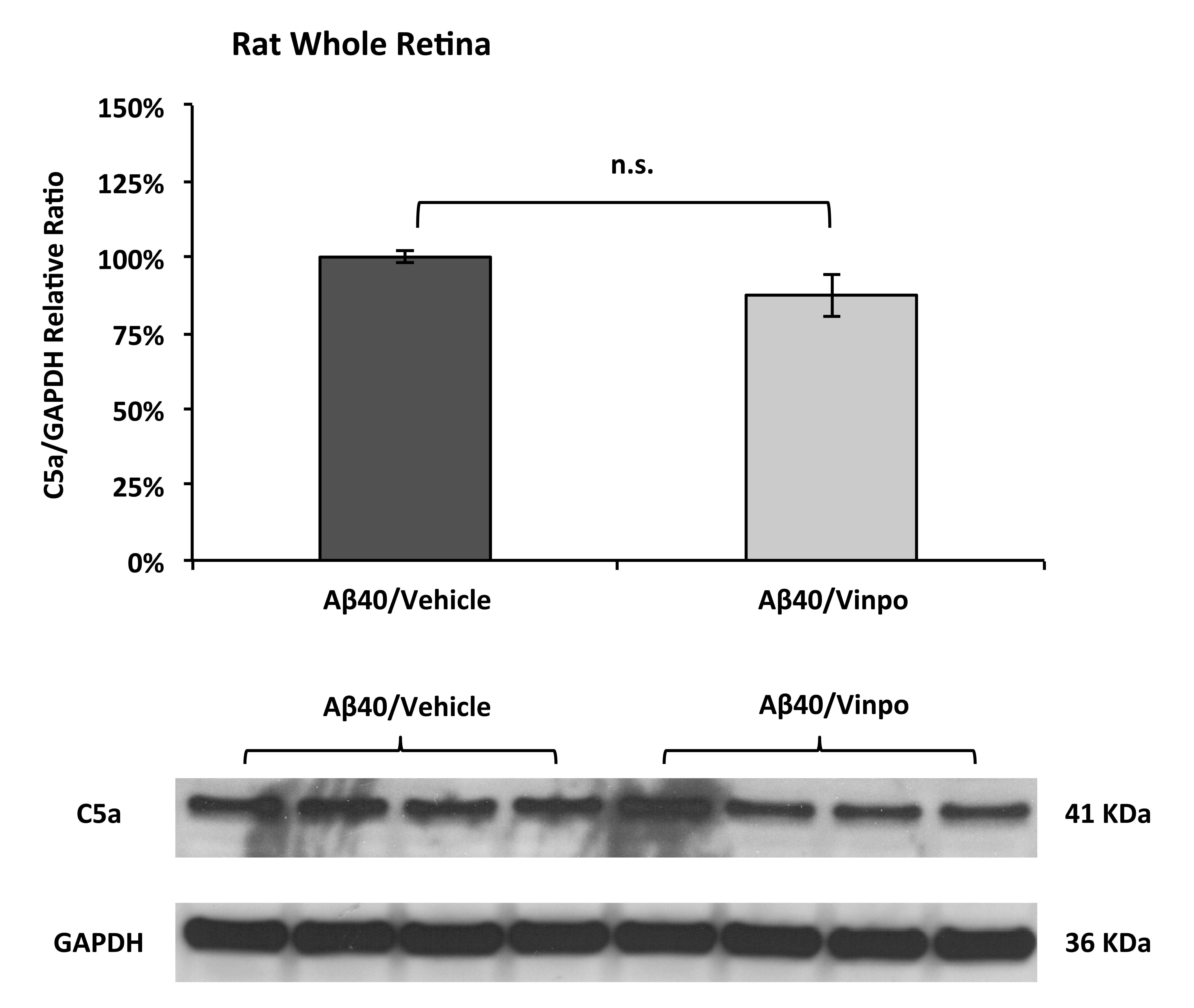

Supplement: Additional file 1: — C5a is not affected by Vinpocetine-mediated NF-κB inhibition. Description of data: Since the intraocular Aβ1–40 injection has been previously implicated in promoting NF-κB activation and NLRP3 inflammasome activation [21], we assessed the level of activated C5 (C5a) as a surrogate marker for complement activation and MAC formation when NF-κB activity was specifically inhibited by vinpocetine. Western blot of retina protein lysates shows equal amounts of C5a (MW 41 KDa; rabbit polyclonal C5a complement antibody, cat# 250565) in the vinpocetine-treated group, when compared to vehicle controls (Mann-Whitney, p > 0.05; N = 5). For a full description of the experimental procedures, readers are referred to our previous publication [28]. [file 12974_2015_337_MOESM1_ESM.jpg]
